# TRIM62 From Chicken as a Negative Regulator of Reticuloendotheliosis Virus Replication

**DOI:** 10.3389/fvets.2020.00152

**Published:** 2020-04-03

**Authors:** Ling Li, Dongyan Niu, Jie Yang, Jianmin Bi, Lingjuan Zhang, Ziqiang Cheng, Guihua Wang

**Affiliations:** ^1^College of Veterinary Medicine, Shandong Agricultural University, Tai'an, China; ^2^Veterinary Medicine, University of Calgary, Calgary, AB, Canada; ^3^China Animal Husbandry Industry Co., Ltd., Beijing, China; ^4^Penglai City Animal Epidemic Prevention and Control Center, Penglai, China

**Keywords:** TRIM62, negative regulation, reticuloendotheliosis virus, domain deletion, RAB5B and ARPC2

## Abstract

Emerging evidence suggests that the tripartite motif containing 62 (TRIM62), a member of the TRIM family, plays an important role in antiviral processes. The objective of the study was to explore the role of TRIM62 in reticuloendotheliosis virus (REV) infection and its potential molecular mechanism. We first demonstrated that the REV infection affected the TRIM62 expression first upregulated and then downregulated in CEF cells. Next, we evaluated the effect of TRIM62 on viral replication. Overexpression of TRIM62 decreased REV replication. On the contrary, silencing of endogenously expressed TRIM62 increased viral replication. Then, to explore the necessity of domains in TRIM62's negative regulation on viral replication, we transfected CEF cells with TRIM62 domain deletion mutants. Deletion domain partially abolished TRIM62's antiviral activity. The effect of SPRY domain deletion was the highest and that of coiled-coil was the lowest. Further, we identified 18 proteins that coimmunoprecipitated and interacted with TRIM62 by immunocoprecipitation and mass spectrometry analysis. Strikingly, among which, both Ras-related protein Rab-5b (RAB5B) and Arp2/3 complex 34-kDa subunit (ARPC2) were involved in actin cytoskeletal pathway. Altogether, these results strongly suggest that chicken TRIM62 provides host defense against viral infection, and all domains are required for its action. RAB5B and ARPC2 may play important roles in its negative regulation processes.

## Introduction

TRIM62 (tripartite motif containing 62) is a member of the TRIM family proteins and is also known as DEAR1 (ductal epithelium-associated RING chromosome) (1). TRIM family proteins, also known as RBCC proteins, contain conserved RING finger, B-box, coiled-coil domains, and a variable C terminus (2). Despite their common domain structure, TRIM proteins play critical roles in distinct cellular processes such as intercellular signaling, innate immunity, transcription, autophagy, and carcinogenesis (3, 4). Members of the TRIM family of E3 ligases exhibit antiviral activities (5, 6). More than 20 TRIM proteins, which affected the entry or release of retrovirus such as human immunodeficiency virus 1 (HIV), murine leukemia virus (MIV), or avian leucosis virus (ALV), were screened (6). Expression of low amounts of TRIM62 enhanced HIV gene expression and release, and the E3 mutant of TRIM62 inhibited HIV release more potently than the wild-type protein (6). However, TRIM62 from orange-spotted grouper (EcTRIM62) negatively regulated the innate antiviral immune response against fish RNA viruses (7).

Reticuloendotheliosis virus (REV) is an avian retrovirus that can induce immunosuppression, runting syndrome, lymphomas, and acute reticulum cell neoplasia (8, 9). The occurrence of reticuloendotheliosis (RE) has an immunosuppressive effect and REV as the contaminant within vaccines against Marek's Disease (MD) (10), fowlpox (11, 12), and Gallid herpesvirus 2 (GaHV-2) (13), which may lead to vaccination failures and co-incidence of RE with other secondary infectious agents. Since breeder and layer flocks are commonly vaccinated against MD, the possible congenital transmission of REV between chickens was also be taking into account. The occurrence of RE has major economic importance. So far, no effective vaccines have been developed against RE; thus, the only protection remains flock renewal with elimination of affected birds or application of experimental antiviral treatment. In a previous study, we have confirmed that TRIM62 possesses restriction of avian leukosis virus subgroup J (ALV-J) replication (14). ALV-J is another avian retrovirus. At present, no data are available regarding the role of TRIM62 from chicken in REV infection.

To explore the role of TRIM62 in REV infection, in the present study, we detected and analyzed the association of TRIM62 expression with viral replication. Then, we evaluated the effects of TRIM62 on viral replication by overexpression, silencing, and domain deletion of TRIM62 in CEF cells with REV infection. Further, with TRIM62 overexpression, we screened key proteins that interacted with TRIM62. Our study provided evidence that chicken TRIM62 negative regulated the REV replication.

## Materials and Methods

### Cell Culture and Viral Infection

The CEF cells' cultural protocol was conducted as described in a previous study (14). CEF cells were incubated with a diluted stock of SNV strain of REV (China strain: JX0927, maintained in our laboratory) at a multiplicity of infection (MOI) of 0.1. The mRNA expression of TRIM62 and REV in CEF cells were detected at different times (12, 24, 48, 72, and 96 h post-infection). The mRNA expression of TRIM62 in the supernatant was also detected in parallel at the same sampling times.

### Plasmid of Chicken TRIM62 and Short Hairpin RNA (shRNA) Transfection

To identify the antiviral function of TRIM62, CEF cells were seeded on six-well-plates for 12 h and transfected with plasmids using a lentiviral vector for TRIM62 overexpression and silence. The transfected plasmids containing chicken TRIM62 (pTRIM62)/domain deletion mutants were for TRIM62 overexpression, and those containing short hairpin RNA targeting TRIM62 (shTRIM62) were for TRIM62 silence. The gene sequence of chicken TRIM62 was obtained from GenBank (XM_015297235.2) (14). These plasmids fused to a Flag tag. The transfected empty vector CEF cells were used as control. The pTRIM62/shTRIM62/pTRIM62-ΔR/B/C/S and empty vector were purchased from Jikai Gene Technology, Inc. (Shanghai, China). After stably expressing TRIM62/shTRIM62 for 12 h/24 h, the transfected CEF cells were incubated with REV. After 72 h, the TRIM62 and viral mRNA/protein levels were detected by qRT-PCR/WB.

### Quantitative Real-Time PCR (qRT-PCR)

Total RNA from CEF cells was isolated using the Tiangen RNeasy mini kit according to the manufacturer's instructions. RNA was reverse transcribed to cDNA using the TaqMan Gold Reverse Transcription kit (Applied Biosystems). qRT-PCR was performed according to a previously described protocol (15, 16). Glyceraldehyde 3-phosphate dehydrogenase (GAPDH) was used as a control for basal RNA levels. Primer sequences are listed in Table 1.

**Table 1:** Primers used for quantitative reverse transcription-PCR.

**Gene target**	**Primer sequence**	**Fragment size (bp)**
TRIM62	Forward: TACTGGGAGGTGGTGGTGTC	246
	Reverse: CGTCGGCGTTGTAGAAGATG	
REV (env)	Forward: TTGTTGAAGGCAAGCATCAG	330
	Reverse: GAGGATAGCATCTGCCCTTT	
RAB5B	Forward: CCCCAGCATCGTCATTG	101
	Reverse: GGCTGTTGTCATCTGCGTAA	
ARPC2	Forward: CGGAAAGGTGTTTATGC	223
	Reverse: CAGGTAGTCTCGGAATGTG	
GADPH	Forward: GAACATCATCCCAGCGTCCA	132
	Reverse: CGGCAGGTCAGGTCAACAAC	

### Western Blotting

The CEF cells were lysed in RIPA lysis buffer [25 mM Tris (pH 7.4), 150 mM NaCl, 1 mM EDTA, 1% NP-40, 5% glycerol] containing protease and phosphatase inhibitor cocktails (Novasygen, Beijing, China). The lysis was separated by SDS-PAGE and transferred to PVDF membranes (Millipore, Bedford, USA) as reported previously (17, 18). The target proteins were detected with specific primary antibodies against TRIM62 (19) and REV env (primary antibodies were prepared by our laboratory, anti-REV gp90) at a 1:200, 1:500, and 1:3,000 dilution, respectively. The secondary antibodies were horseradish peroxidase (HRP)-conjugated enhanced chemiluminescence (ECL) goat. The blots were visualized by the ECL-enhanced chemiluminescence kit (Roche, Basel, Switzerland).

### Immunocoprecipitation

According to the instructions of Pierce Co-Immunoprecipitation (Co-IP) Kit (Thermo, Thermo Fisher Scientific, Massachusetts, USA), the 12-h pTRIM62 transfected CEF cells were subjected to REV infection for 72 h. The REV-infected CEF cells were then harvested and lysed. The anti-Flag label antibody was incubated with AminoLink coupling resin for 2 h at room temperature for antibody immunobilization. After centrifugation of cell lysates, supernatants were immunoprecipitated with coupling antibody. After another centrifugation of immunoprecipitated supernatants, the protein complex was washed three times with RIPA lysis buffer. Immunoprecipitates were incubated with elution buffer for 5 min at room temperature and centrifuged. The prey complex was collected and stored at −80°C. CEF cells were transfected with empty vector infected with REV as control.

### Liquid Chromatography-Mass Spectrometry Analysis

Ten micrograms of the above prey complex was incubated in SDS-PAGE sample buffer (Solarbio, Beijing, China) for 5 min at 95°C. Proteins of cell lysates were separated by SDS-PAGE gels for 10 min and formed into a line. Each of the SDS-PAGE gel lanes was cut and subjected to trypsin digestion. Mass spectrometry (MS) analysis was performed according to a previously reported protocol (20) for detecting polypeptide sequence of proteins. Independent triplicate samples were analyzed. Polypeptide sequence was identified against using the Gallus (chicken) database and viral database (uniport-Gallusgalluschicken_−_REV_−_Combined.fasta) using Proteome Discoverer 1.4 (Thermo Fisher Scientific, Massachusetts, USA) software.

### Statistical Analysis

Results are presented as the means ± standard deviations (SD) of at least three sample replicates. Statistical analysis was performed using SPSS 19.0 statistical software, and *p* < 0.05 was considered statistically significant.

## Results

### REV Infection Affected TRIM62 Expression in CEF Cells

**Figure 1:**
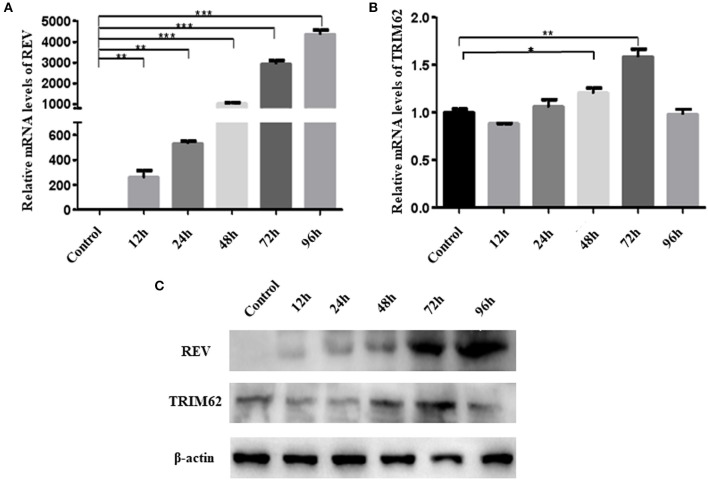
Effect of REV infection on TRIM62 expression. CEF cells were infected with REV. The RNA expression levels of REV **(A)** and of TRIM62 **(B)** at 12, 24, 48, 72, and 96 h post-infection in CEF cells were assessed by qRT-PCR. **(C)** The protein expression levels of REV and TRIM62 were detected by WB. Uninfected CEF cells were used as the control. *p < 0.05, **p < 0.01, and ***p < 0.001.

To assess association between the expression of TRIM62 and REV, we measured the TRIM62 expression and REV replication in REV-infected CEF cells at the transcriptional and translational level. Time course infection of REV in CEF cells showed that the REV mRNA levels were significantly increased (*p* < 0.001) from 48 to 96 h post-infection (Figure 1A), and the TRIM62 mRNA levels were also significantly upregulated at 48 h (*p* < 0.1) and 72 h (*p* < 0.1) as compared to that in uninfected CEF cells. However, compared with control, there were no changes of TRIM62 mRNA levels at another time point (Figure 1B). The REV mRNA expression was upregulated and reached the plateau at 96 h post-infection in CEF cells (17).

The protein levels of REV and TRIM62 were detected by WB, which were consistent with the dynamic changes of mRNA levels. Compared with control, the viral protein levels were significantly increased (*p* < 0.001) from 48 to 96 h post-infection, and TRIM62 protein levels were also significantly upregulated at 48 and 72 h (*p* < 0.01). However, there were no changes of TRIM62 protein levels at another time point observed (Figure 1C).

### TRIM62 Restricted REV Replication in CEF Cells

**Figure 2:**
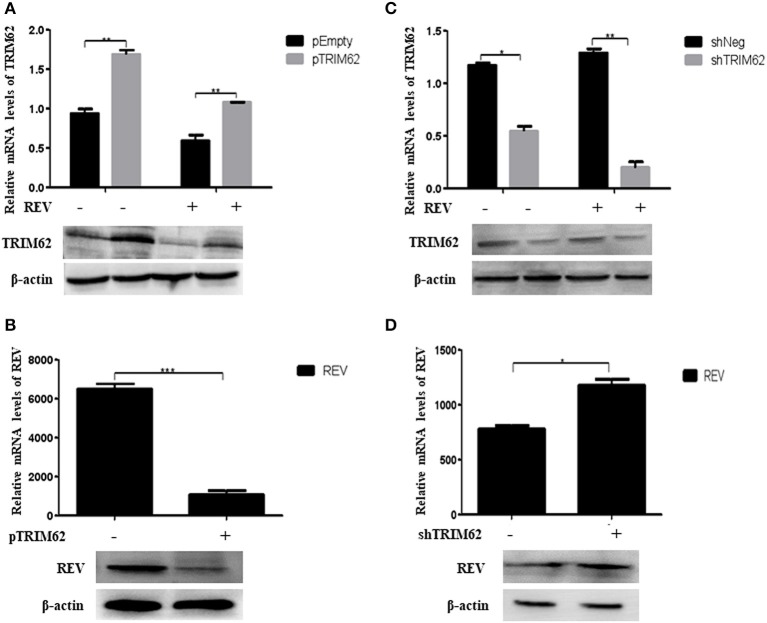
Restriction of REV replication in CEF cells induced by TRIM62. **(A,B)** CEF cells were transfected with pTRIM62 or empty vector before infection with REV. The expression levels of TRIM62 **(A)** and REV **(B)** were assessed by qRT-PCR and Western blotting. **(C,D)** CEF cells were transfected with shTRIM62 or a negative-control shRNA before infection with REV. The expression levels of TRIM62 **(C)** and REV **(D)** were determined by qPCR and Western blotting. *p < 0.05, **p < 0.01, and ***p < 0.001.

We overexpressed or silenced TRIM62 to detect the role of TRIM62 in REV replication in infected CEF cells. Compared with non-transfected cells, despite REV infection, the TRIM62 mRNA level was significantly greater (*p* < 0.01) in pTRIM62-transfected CEF cells (Figure 2A) and lower (*p* < 0.01) in shTRIM62-transfected CEF cells (Figure 2C). The TRIM62 overexpression decreased the REV mRNA expression in transfected cells (Figure 2B), whereas silence of TRIM62 increased the mRNA expression of REV (Figure 2D). These results were confirmed in protein levels by Western blotting (Figures 2A–D). Our results strongly suggest the role of TRIM62 in restricting REV infection.

### Domain Deletion-Induced TRIM62's Antiviral Activity Abolished Partially

**Figure 3:**
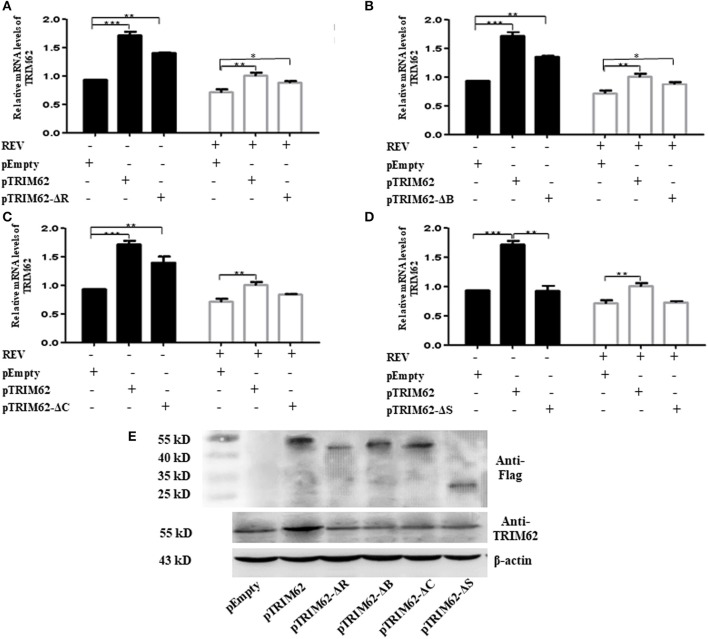
Effect of domain deletion on TRIM62 expression. The relative mRNA expression of TRIM62 in CEF cells transfected with RING **(A)**, B-box **(B)**, coiled-coil **(C)**, or SPRY **(D)** domain deletion mutant with or without REV infection were measured by qRT-PCR using pEmpty and pTRIM62 as controls. **(E)** TRIM62 protein expression levels were detected with specific primary antibodies against Flag label and TRIM62 by Western blotting, respectively. pEmpty represent empty vector, pTRIM62 represent TRIM62 full length, pTRIM62-ΔR represent RING domain deletion, pTRIM62-ΔB represent B-box domain deletion, pTRIM62-ΔC represent coiled-coil domain deletion, and pTRIM62-ΔS represent SPRY domain deletion. *p < 0.05, **p < 0.01, and ***p < 0.001.

To further investigate the effect of domain on negative regulation of TRIM62, we explored the effect of TRIM62 domain deletion mutants on TRIM62 expression and REV replication. The mutants were prepared as previously described (14). Regardless of REV infection, the expression of TRIM62 was significantly higher in cells transfected with deletion of RING (Figure 3A) and B-box (Figure 3B) than empty vector (*p* > 0.1). When transfected with coiled-coil domain deletion mutant, viral infection decreased the expression of TRIM62 in cells (Figure 3C). These results indicated that the effect of REV infection on TRIM62 may associate with the coiled-coil domain. Compared with empty vector, there was no difference obtained of TRIM62 expression in cells transfected with deletion of SPRY domain mutant (Figure 3D). The qRT-PCR primers located in the SPRY domain may explain this result that deletion of SPRY domain did not result in difference of TRIM62 mRNA expression. To further confirm the results, we measured the TRIM62 protein expression with specific primary antibodies against TRIM62 (Figure 3E). The expression of TRIM62 in mutant-transfected cells is lower than that in complete TRIM62-transfected cells.

**Figure 4:**
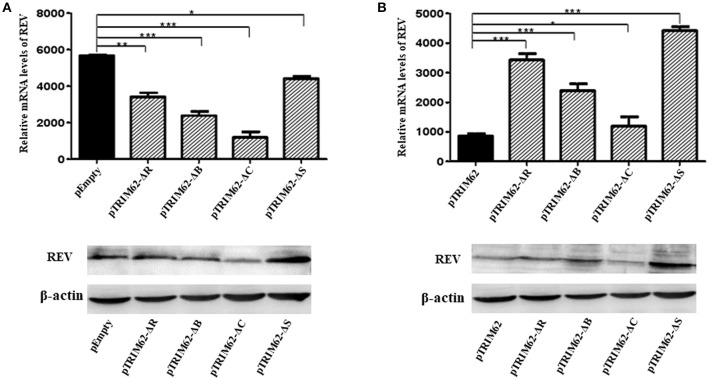
Inhibition of REV RNA expression in CEF cells by TRIM62 RING, B-box, coiled-coil, and SPRY domain deletion mutants. The relative mRNA expression levels of REV in CEF cells transfected with RING, B-box, coiled-coil, or SPRY domain deletion mutants were measured by qRT-PCR and Western blotting. pEmpty **(A)** and pTRIM62 **(B)** were used as controls. pEmpty represent empty vector, pTRIM62 represent TRIM62 full-length, pTRIM62-ΔR represent RING domain deletion, pTRIM62-ΔB represent B-box domain deletion, pTRIM62-ΔC represent coiled-coil domain deletion, and pTRIM62-ΔS represent SPRY domain deletion. *p < 0.05, **p < 0.01, and ***p < 0.001.

As compared with pEmpty vector-transfected cells, the level of REV mRNA expression was significant lower (*p* < 0.1) in CEF cells transfected by any of the TRIM62 domain deletion mutants (Figure 4A). The effect of coiled-coil domain deletion was the highest (77.9 ± 2.3%), and that of SPRY domain deletion was the lowest (22.8 ± 2.5%). Compared with complete TRIM62, the mRNA expression of REV was significantly higher in cells transfected with domain deletion mutants (Figure 4B). The viral expression in SPRY domain deletion-transfected cells was the highest (435.5 ± 6.2%), and that in coiled-coil domain deletion-transfected cells was the lowest (53.2% ± 6.4%). These results suggested that the deletion of domains partially abolished the antiviral activity of TRIM62.

### Identification of Cell Proteins That Interact With TRIM62

**Figure 5:**
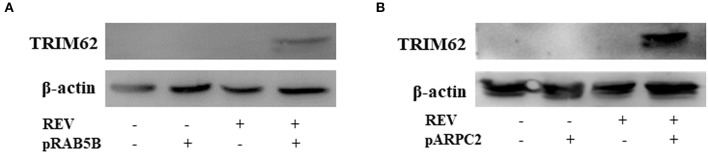
Validation of the interaction of TRIM62 with ARB5B and ARPC2. CEF cells were transfected with a plasmid expression Flag-tagged RAB5B **(A)** and APRC2 **(B)** before REV infection/uninfection and immunoprecipitated with anti-Flag antibody. The TRIM62 in immunoprecipitates was detected by Western blotting with anti-TRIM62 antibody.

The REV infection affected the TRIM62 expression first upregulated and then downregulated in CEF cells. We hypothesized that other important interacting proteins besides the domains were involved in TRIM62's negative regulation of REV replication. To identify host cell proteins that interact with TRIM62, we used a tandem affinity purification approach coupled with mass spectrometry-based proteomics technology. These experiments identified 18 cell proteins that coimmunoprecipitated with transiently expressed Flag-tagged TRIM62 (Table 2). Decades of HIV research have testified to the integral role of the actin cytoskeleton in both establishing and spreading the infection (21). Of the TRIM62-interacting cell proteins identified, we focused on cytoskeletal proteins RAB5B and ARPC2. To verify the interaction between TRIM62 and RAB5B/ARPC2, CEF cells were transfected with a plasmid expression Flag-tagged RAB5B/APRC2 before REV infection, immunoprecipitated with anti-Flag antibody, and immunoblotted with anti-TRIM62 antibody. CEF cells were transfected with plasmid or empty vector uninfected with REV and transfected with empty vector infected with REV as control, respectively. Upon REV infection, anti-TRIM62 coprecipitated Flag-tag RAB5B (Figure 5A) and Flag-tag ARPC2 (Figure 5B).

**Table 2:** Identified proteins interacting with TRIM62.

**No**.	**Accession**	**Gene**	**Protein**	**Coverage**
1	A0A1L1RRL2	ACTB	Actin, cytoplasmic 1	30.12
2	A0A1L1RSN4	ACTC1	Actin, alpha cardiac muscle 1	25.61
3	F1NJ08	VIM	Vimentin	17.39
4	E1C2H4	LEMD2	LEM domain containing 2	4.79
5	A0A1L1RWD5	RPL15	Ribosomal protein L15	17.16
6	F1NSP8	HNRNPU	Heterogeneous nuclear ribonucleoprotein U	1.78
7	A0A1D5PW24	RPL19	Ribosomal protein L19	14.05
8	P01994	HBAA	Hemoglobin subunit alpha-A	10.56
9	A0A1L1RLB1	PKM	Pyruvate kinase PKM	5.81
10	**A0A1D5PKU6**	**RAB5**	**Ras-related protein Rab-5**	**5.14**
11	Q05744	CTSD	Cathepsin D	4.52
12	**A0A1L1RPQ9**	**Arp2/3**	**Arp2/3 complex 34 kD subunit**	**3.90**
13	A0A1L1RM78	PPIA	Peptidyl-prolyl cis-trans isomerase	12.00
14	A0A1D5NYW5	N/A	Peptidylprolyl isomerase	7.35
15	A0A1D5PZE3	APOA1	Apolipoprotein A-I	5.70
16	F1NK96	PDIA6	Protein disulfide isomerase family A member 6	6.04
17	A0A1D5PJM6	NUP214	Nucleoporin 214	0.59
18	F1NWX0	LOC425049	Tubulin alpha chain	6.46

*The bold means the two proteins are involved in the skeleton pathway*.

**Figure 6:**
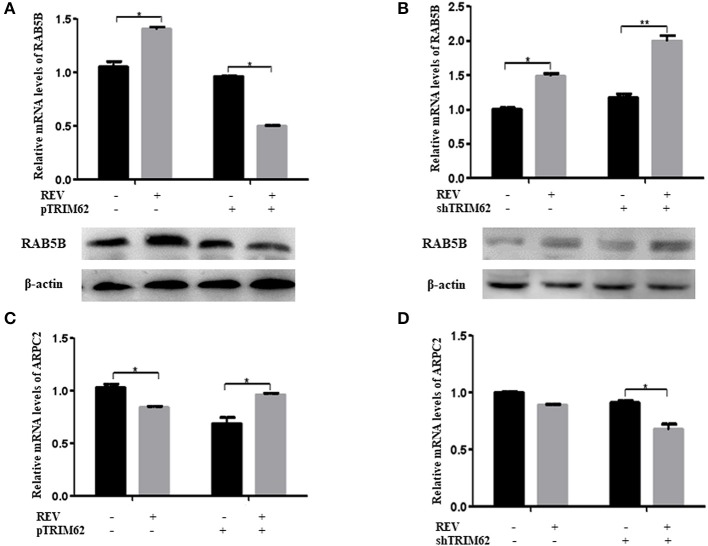
Effect of TRIM62 on the expression of RAB5B and ARPC2. **(A,B)** The relative mRNA and protein expression of RAB5B and in CEF cells were transfected with pTRIM62 **(A)** or shTRIM62 **(B)**. **(C,D)** The relative mRNA expression of ARPC2 in CEF cells were transfected with pTRIM62 **(C)** or shTRIM62 **(D)**. *p < 0.05, **p < 0.01.

Further, we found that TRIM62 affected the expression of RAB5B and ARPC2. As shown in Figures 6A,B, in CEF cells infected with REV, overexpression of TRIM62 exhibited a decrease of RAB5B expression, and silencing of TRIM62 enhanced the expression of RAB5B. On the contrary, in CEF cells infected with REV, the overexpression of TRIM62 resulted in an increase of ARPC2 expression, and silencing of TRM62 reduced the expression of ARPC2 (Figures 6C,D). These results indicated that RAB5B and ARPC2 play important roles in the negative regulation of TRIM62 on REV replication.

## Discussion

The host innate immune system senses antigen and elicits local antiviral defense to control infection. However, the REV can evade surveillance by the immune system after infection. TRIM62 is an innate immune regulator (6). In this study, we found that the TRIM62 expression levels were first up and then down with the increase of REV replication in CEF cells. These results suggest that as an innate immune factor, TRIM62 was activated and induced a short periodic significant upregulation at the fast replication period of REV (*p* < 0.001), and REV may circumvent TRIM62's restrictive effects by reducing its expression levels. However, we did not obtain valid data of TRIM62 expression in supernatant because the RNA concentration was too low. The reason that TRIM62 expression was first increased and then decreased needed further confirmation.

In the current study, TRIM62 has been identified as a protein that negatively regulates REV replication. Gene silencing of TRIM62 enhanced REV infection, which indicated that TRIM62 contributes to the endogenous restriction of REV in CEF cells. The further characterization of mechanisms by which REV reduces TRIM62 expression is warranted.

Owing to the presence of similar domains, the TRIM proteins were involved in similar antiviral biological processes. However, the domains of TRIM proteins played complicated roles in the inhibition of different viruses. Deletion of the PRY-SPRY domain of TRIM62 from human did not compromise its signal transduction properties. Rather, in addition to the RING domain, both B-box and coiled-coil domains were required for NF-κB and AP-1 induction by TRIM62 (5). Thus, the B-box and/or coiled-coil domains that confer oligomerization/dimerization properties (22) are indispensable for TRIM62-mediated signaling (5). The SPRY domain mediated the affinity for the viral capsid and the function of RING domain to TRIM5α restriction of retrovirus infection was determined by the target virus (23). The antiviral activity of fish TRIM36 required both RING and SPRY domains (24). The RING and C-terminal tail were essential for TRIM56's antiviral activity against flaviviruses (25). Interestingly, the inhibitory effect of fish TRIM62 on interferon immune and inflammation response to negatively regulate virus replication was also dependent on RING and SPRY domains (7). In our study, this varied expression level of mutants (Figure 3) could be due to variation in transfection and expression efficiency of different plasmids in primary cells. Even though our results demonstrated that RING, B-box, coiled-coil, and SPRY domains contribute to the antiviral activity of chicken TRIM62, the effect of RING and SPRY was more significant than that of B-box and coiled-coil (Figures 4A,B). These results indicated that RING and SPRY domains are related to the antiviral activity of TRIM proteins.

Retroviruses are considered to use vesicular trafficking in infected cells (18, 26, 27). RAB5B is an isoform of RAB5, which is a member of the RAB family, a small GTPase family. RAB5B regulates fusion and motility of early endosomes, and is a marker of the early endosome compartment (28). RAB5B is a major regulator of hepatitis B virus (HBV) production (29). APRC2 is a component of Arp2/3 complex. The T cell-specific deletion of ARPC2 results in compromised peripheral T cell homeostasis (30). T cell survival and proliferation are mediated by complex homeostatic signals. Peripheral T cells are maintained at a constant cell number so that they can efficiently recognize foreign antigens and protect the host from pathogen invasion (31). Thus, of the TRIM62-interacting cell proteins identified, we focused on RAB5B and ARPC2. The interaction between RAB5B/ARPC2 and TRIM62 indicated that they played important roles in TRIM62 negative regulation on REV replication. A detailed understanding of host restriction may lead to antiviral therapies aimed at strengthening the innate immunity to retroviruses at the cellular and molecular level.

In addition, TRIM62 is a putative tumor suppressor and the TRIM62 levels represent an important prognostic marker in lung tumor (32) acute myeloid leukemia (AML) (33) and cervical cancer (34). ARPC2 promotes proliferation and metastasis (35, 36). REV is an oncogenic retrovirus. Further study is warranted to investigate the potential function of chicken TRIM62 and ARPC2 on REV infection inducing tumor formation.

## Conclusion

We demonstrated that TRIM62 is a new suppressor that negatively regulates REV replication. The deletion of RING, B-box, coiled-coil, and SPRY domains partially affected TRIM62's antiviral activity, and the effects of RING and SPRY deletion were more significant than that of B-box and coiled-coil. The results suggested that all domains, RING, B-box, coiled-coil, and SPRY domains, are required for chicken TRIM62 to provide host defense against viral replication. Furthermore, both RAB5B and ARPC2 interacted with TRIM62, and maybe played important roles in TRIM62 negative regulation on REV replication. Our study provided a potential antiviral strategy targeting this novel regulator.

## Data Availability Statement

The datasets generated for this study are available on request to the corresponding author.

## Author Contributions

GW designed the experiments. LL, DN, and JY performed the experiments. GW, LL, and DN prepared the manuscript. JB, LZ, and ZC analyzed the data. All authors have read and approved the final version of the manuscript.

### Conflict of Interest

JB was employed by the company China Animal Husbandry Industry Co., Ltd. The remaining authors declare that the research was conducted in the absence of any commercial or financial relationships that could be construed as a potential conflict of interest.
